# Adherence to a Smartphone Application for Weight Loss Compared to Website and Paper Diary: Pilot Randomized Controlled Trial

**DOI:** 10.2196/jmir.2283

**Published:** 2013-04-15

**Authors:** Michelle Clare Carter, Victoria Jane Burley, Camilla Nykjaer, Janet Elizabeth Cade

**Affiliations:** ^1^Nutritional Epidemiology GroupSchool of Food Science and NutritionUniversity of LeedsLeedsUnited Kingdom

**Keywords:** smartphone, obesity, text message, app

## Abstract

**Background:**

There is growing interest in the use of information communication technologies to treat obesity. An intervention delivered by smartphone could be a convenient, potentially cost-effective, and wide-reaching weight management strategy. Although there have been studies of texting-based interventions and smartphone applications (apps) used as adjuncts to other treatments, there are currently no randomized controlled trials (RCT) of a stand-alone smartphone application for weight loss that focuses primarily on self-monitoring of diet and physical activity.

**Objective:**

The aim of this pilot study was to collect acceptability and feasibility outcomes of a self-monitoring weight management intervention delivered by a smartphone app, compared to a website and paper diary.

**Methods:**

A sample of 128 overweight volunteers were randomized to receive a weight management intervention delivered by smartphone app, website, or paper diary. The smartphone app intervention, My Meal Mate (MMM), was developed by the research team using an evidence-based behavioral approach. The app incorporates goal setting, self-monitoring of diet and activity, and feedback via weekly text message. The website group used an existing commercially available slimming website from a company called Weight Loss Resources who also provided the paper diaries. The comparator groups delivered a similar self-monitoring intervention to the app, but by different modes of delivery. Participants were recruited by email, intranet, newsletters, and posters from large local employers. Trial duration was 6 months. The intervention and comparator groups were self-directed with no ongoing human input from the research team. The only face-to-face components were at baseline enrollment and brief follow-up sessions at 6 weeks and 6 months to take anthropometric measures and administer questionnaires.

**Results:**

Trial retention was 40/43 (93%) in the smartphone group, 19/42 (55%) in the website group, and 20/43 (53%) in the diary group at 6 months. Adherence was statistically significantly higher in the smartphone group with a mean of 92 days (SD 67) of dietary recording compared with 35 days (SD 44) in the website group and 29 days (SD 39) in the diary group (*P*<.001). Self-monitoring declined over time in all groups. In an intention-to-treat analysis using baseline observation carried forward for missing data, mean weight change at 6 months was -4.6 kg (95% CI –6.2 to –3.0) in the smartphone app group, –2.9 kg (95% CI –4.7 to –1.1) in the diary group, and –1.3 kg (95% CI –2.7 to 0.1) in the website group. BMI change at 6 months was –1.6 kg/m^2^ (95% CI –2.2 to –1.1) in the smartphone group, –1.0 kg/m^2^ (95% CI –1.6 to –0.4) in the diary group, and –0.5 kg/m^2^ (95% CI –0.9 to 0.0) in the website group. Change in body fat was –1.3% (95% CI –1.7 to –0.8) in the smartphone group, –0.9% (95% CI –1.5 to –0.4) in the diary group, and –0.5% (95% CI –0.9 to 0.0) in the website group.

**Conclusions:**

The MMM app is an acceptable and feasible weight loss intervention and a full RCT of this approach is warranted.

**Trial Registration:**

ClinicalTrials.gov NCT01744535; http://clinicaltrials.gov/ct2/show/NCT01744535 (Archived by WebCite at http://www.webcitation.org/6FEtc3PVB)

## Introduction

Obesity is estimated by the World Health Organization to be the fifth leading risk for global deaths [1], and it is associated with a range of serious and difficult to treat conditions, such as diabetes, some cancers, and heart disease. In the United Kingdom, obesity is a major public health concern reported to affect a quarter of the adult population [2]. The economic burden to the National Health Service (NHS) is significant, with the direct cost of spending on overweight and obesity estimated at £4.2 billion in 2007 [3]. The effective treatment of obesity and overweight is challenging and NHS primary care struggles to provide effective support to meet demand [4]. A large community-based survey showed that individuals desire alternatives to face-to-face weight loss treatments and, if given the choice, some would be interested in engaging with minimal contact weight management programs [5].

Weight management interventions based on information and communication technology (ICT) provide an opportunity to engage a wide audience in a potentially flexible and cost-effective way. In recent years, research into mobile devices to facilitate dietary and physical activity self-monitoring and weight-related behavior change has grown. Mobile phones, in particular, are an intuitively appealing intervention platform given that they are ubiquitous, engaging, and portable [6]. Researchers have investigated text message short message service (SMS) interventions to promote change in diet [7-9] and physical activity [10,11]. For example, in a small randomized controlled trial (RCT) (N=75) an SMS intervention lasting 16 weeks led to a mean weight loss of 2.1 kg (95% CI -3 to -1) in a group receiving daily text messages compared to 0.4 kg (95% CI -1 to 1) in the control group [7]. However, in a follow-up study, a larger 12-month RCT of an SMS intervention in 170 overweight and obese adults showed no statistically significant difference in weight loss between the intervention and control group [12]. In that study, adherence to the text-messaging intervention was found to be related to greater weight loss and the authors concluded that text messages might be a useful adjunct to a weight loss program. Researchers have also investigated dietary self-monitoring-based electronic interventions using personal digital assistants (PDAs), electronic portable devices that share some of the features of mobile phones. A 6-month RCT compared a PDA alone and a PDA with feedback to a paper diary in a sample of 210 overweight adults. The PDA group (combined with feedback) had the highest proportion of participants achieving greater than 5% weight loss (63% compared with 46% in the PDA alone and 49% in the food diary alone) after 6 months [13].

A number of smartphone applications (apps) that use the computational abilities of the phone for self-monitoring rather than just the SMS component have been developed (14-17) and tested. For example, a mobile app developed for a Nokia platform, Wellness Diary, allows users to record health-related data such as weight, sleep, and physical activity, and receive feedback on input [14-16]. The Patient-Centered Assessment and Counseling Mobile Energy Balance (PmEB) app allows users to log food intake from a limited database of foods and track calorie balance [18]. Another app, UbiFit, was developed to promote change in physical activity [19]. However, none of these apps have been formally evaluated in an RCT. In a recent 6-month RCT, 96 overweight and obese participants were randomized to either a group receiving podcasts only or an enhanced group using the podcasts, Twitter, and a smartphone app called FatSecret for self-monitoring [20]. In this study, the enhanced group were not found to have greater weight loss than the podcast-only group.

We developed a smartphone app for weight loss called My Meal Mate (MMM). The enhanced computational ability of a smartphone allows detailed self-monitoring (of diet, physical activity, and weight) and feedback via text message to be combined into 1 intervention. The MMM app uses an Android operating system so it can be trialed on an up-to-date and popular handset. The app has been benchmarked against commercially available systems, such as MyFitnessPal [21], and contains a large, detailed UK-branded food database [22]. These factors are important to engage users with the app in a real-life setting. Although there have been RCTs using text-messaging interventions for weight management, PDAs for self-monitoring and smartphone apps as adjuncts to other weight management interventions, to our knowledge there have not been any RCTs of a smartphone app as a weight loss intervention in itself using both self-monitoring and text-messaging functions. A trial of this type is necessary because smartphone apps are readily available to the public to download and likely to be used as a stand-alone intervention rather than as an adjunct to another intervention (such as podcasts or face-to-face advice). The aim of this pilot was to test the acceptability and feasibility (recruitment, dropout, and adherence) of MMM with a view to informing a larger trial.

## Methods

### Recruitment Strategy

Participants were recruited from large employers within Leeds, United Kingdom, by advertising through email, intranet, posters, and newsletters. Advertising material encouraged participants to contact the research team, following which they were emailed information sheets and an eligibility questionnaire. The eligibility criteria was a body mass index (BMI) of ≥27 kg/m^2^; aged 18 to 65 years; willing to commit the necessary time and effort to the study; employed by a large employer in Leeds; not pregnant, breast-feeding, or planning a pregnancy; not taking antiobesity medication or medication/insulin for diabetes; never had surgery for weight loss; not taking the antidepressant sertraline (due to associations with weight gain); able to read and write in English; able to access the Internet; and willing to be randomized to 1 of 3 groups. An inclusion cutoff BMI of ≥27 kg/m^2^, as opposed to the more familiar cutoff point of 25 kg/m^2^, was chosen to ensure that participants had a reasonable amount of weight to lose in 6 months before maintenance of weight loss and also as a safety measure so that they would be unlikely to lose so much weight that they fell below the defined healthy BMI range given that the app would be used for 6 months without any clinical supervision.

### Interventions

**Figure 1:**
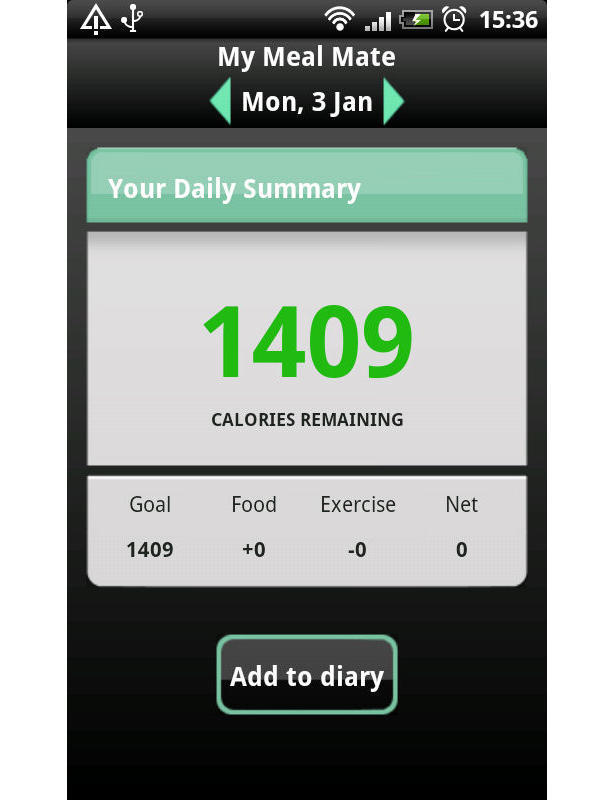
Screenshot of the My Meal Mate (MMM) homepage.

**Figure 2:**
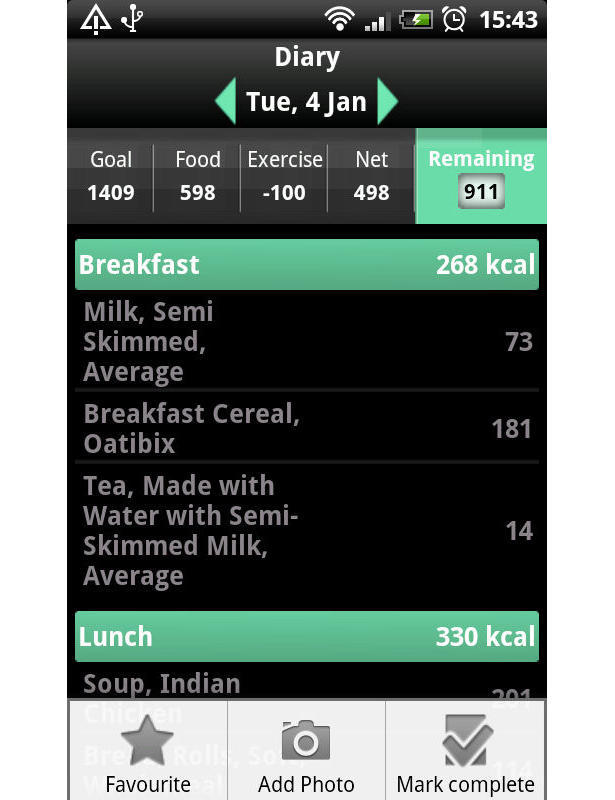
Screenshot of the My Meal Mate (MMM) food diary page.

The researchers developed the MMM smartphone app for weight loss to be used on an Android operating system. Figures 1 and 2 are screenshots of the app. During development of MMM, several commercially available systems, such as MyFitnessPal and Calorie Counter, were informally evaluated by the researchers and by discussion in focus groups with potential system users. The MMM app was benchmarked in this way to produce an app of equivalent appearance and functionality as other apps available to the general public to download. Current UK evidence-based obesity guidelines advocate a lifestyle change approach to treatment [23]; therefore, in-line with this, the key behavioral strategies of goal setting, self-monitoring, and feedback underpin the MMM app. The MMM app allows users to set a weight loss goal and self-monitor daily calorie intake toward achieving that goal. Users select the food and drink consumed from a database and log items in an electronic food diary. Physical activity can also be recorded in the diary enabling the user to receive instant feedback on their energy expenditure. Progress is tracked graphically and further support is provided through tailored weekly text messages. A library of text messages was created and each message was triggered according to progress toward the users’ calorie targets. The messages aimed to enhance the users’ self-efficacy by encouraging the users to rehearse their weight loss goals and reinforce positive behavioral beliefs (about competence, confidence, and mastery). The MMM app has several usability features, such as the ability to take photographs of food to serve as a memory aid, and store favorite meal combinations and recently used items. The app has an associated Web interface to upload the recorded data. A unique feature of MMM is the large UK-specific branded food database which was provided by Weight Loss Resources, a commercial company [22]. The database contains 23,000 food and drink records that reflect both generic and branded items. The diet measures captured on MMM have been found to correlate well with a reference measure of diet [24]. There are a series of YouTube videos which give a detailed account of each feature of the MMM app that participants were able to directly link to for help [25].

The MMM app was compared against 2 other self-monitoring interventions to allow comparison of self-monitoring on a mobile phone against other approaches. The comparison groups used either a self-monitoring slimming website [22] or a food diary accompanied by a calorie-counting book [26]. The comparison interventions provided an opportunity to deliver a similar self-monitoring intervention by different mediums because each provides goal setting and self-monitoring by using the same Weight Loss Resources food database.

### Procedure

The trial design was a 3-armed parallel group randomized trial. As a pilot trial, the primary outcomes were feasibility and acceptability outcomes of adherence to the trial and adherence to the interventions (frequency of use). Secondary outcomes were anthropometric, which were objectively measured to give an idea of effect size. Eligible participants were invited to attend a baseline enrollment session at the University of Leeds where height, weight, and percentage body fat (BF) were measured by research assistants, and baseline questionnaires self-completed. The questionnaires were designed to collect information on demographics, technology usage, attitudes toward weight loss, physical activity [27], eating behavior [28], and a variety of psychosocial variables [29,30]. Weight (without shoes) and BF were measured by using Weight Watchers 8958U Body Analyser Scale portable weighing scales. Height (without shoes) was measured using a portable stadiometer to the nearest 0.1 cm. After measurements were taken, participants were randomized by a process of minimization using the Minim software package [31] to 1 of 3 groups. The minimization balanced equally at the medians on 3 factors: starting BMI, age, and gender. Minimization was used because this method has the advantage over simple or stratified randomization of providing very similar balanced groups in small samples [32].

After randomization, groups of participants were taken to separate rooms to receive standardized training in their allocated study equipment. Participants were instructed to use the study equipment every day for a week and then to use it as much as they desired over the trial period. The smartphone group were given a HTC Desire smartphone with the MMM app predownloaded, the website group were given a voucher providing 6 months access to the Weight Loss Resources website, and the food diary group were given a paper food diary, a calorie-counting book, and a calculator. All participants were given access to an Internet forum for social support. The baseline enrollment sessions took place over the month of June 2011 with participants enrolling in small groups at a time. Participants returned for repeat measures at 6 weeks and 6 months after randomization. Evaluation questionnaires were also administered at 6 weeks and 6 months. At 6 months, study equipment was returned. Because of the nature of the interventions, it was not possible to blind participants to their assignment. Fieldworkers carrying out measurements on participants were blinded to group assignment and participants were asked not to discuss their group allocation when measurements were taken.

### Sample Size Determination

This was not a phase III trial; therefore, a formal sample size calculation was not appropriate and there are few published guidelines on recommended sample sizes for pilot trials. The trial aimed to recruit a sample size of 135, which was a pragmatic decision based partly on the amount of available study equipment.

### Statistical Analysis

Statistical analysis was carried out using Stata Statistical Software Release 11 (StataCorp LP, College Station, TX, USA). Most analyses are descriptive because this was a pilot trial and not powered to detect weight change. The effectiveness of the minimization procedure was assessed by determining baseline balance among the groups. When analyzing differences among the 3 intervention groups, 1-way analysis of variance (ANOVA) was used for continuous outcomes found to be normally distributed or the Kruskal-Wallis test when data were not normally distributed. For the analysis of completers versus noncompleters, *t* tests were used for continuous outcomes that were normally distributed and the Wilcoxon rank sum test for non-normally distributed outcomes. Differences among groups for categorical data were analyzed by using Chi-square tests.

This pilot trial was not statistically powered to detect change in anthropometric measures; however, results are displayed for interest and to provide information on effect size. A regression analysis was used to test between-group differences in change in anthropometric measures adjusting for the 3 factors used in randomization at baseline (age, gender, and starting BMI). Two analyses were conducted because there was a proportion of missing data and unequal dropout between groups: an intention-to-treat analysis in which all are included but using baseline weight carried forward for missing data, and an analysis in just those who completed 6-month follow-up.

### Ethical Approval

This trial was conducted according to the guidelines laid down in the Declaration of Helsinki and all procedures involving human subjects were approved by the University of Leeds, Faculty of Medicine and Health Research ethics committee (ethics reference no: HSLTLM/10/002). Written informed consent was obtained from all trial participants. In accordance with the requirements of the International Committee of Medical Journal Editors (ICMJE), this trial was registered (ClinicalTrials.gov NCT01744535) and reported in accordance with the CONSORT-EHEALTH checklist [33]. The version number of the app tested in the pilot trial was 1.0.23.

## Results

### Baseline Characteristics

Table 1 shows the baseline characteristics of the pilot study participants by group. There were no statistically significant differences found among the 3 intervention groups for the factors balanced at minimization: gender (*P*=.97), age (*P*=.82), and BMI (*P*=.74). Of the 128 adults enrolled, 77% (99/128) were female and 91% (117/128) were white. The mean age of participants was 42 years (SD 9) and over half (58%, 74/127) were employed in managerial and professional occupations. The mean participant BMI was 34 kg/m^2^ (SD 5) with over three-quarters of participants (77%, 98/128) classified as obese (BMI≥30 kg/m^2^).

### Recruitment

**Figure 3:**
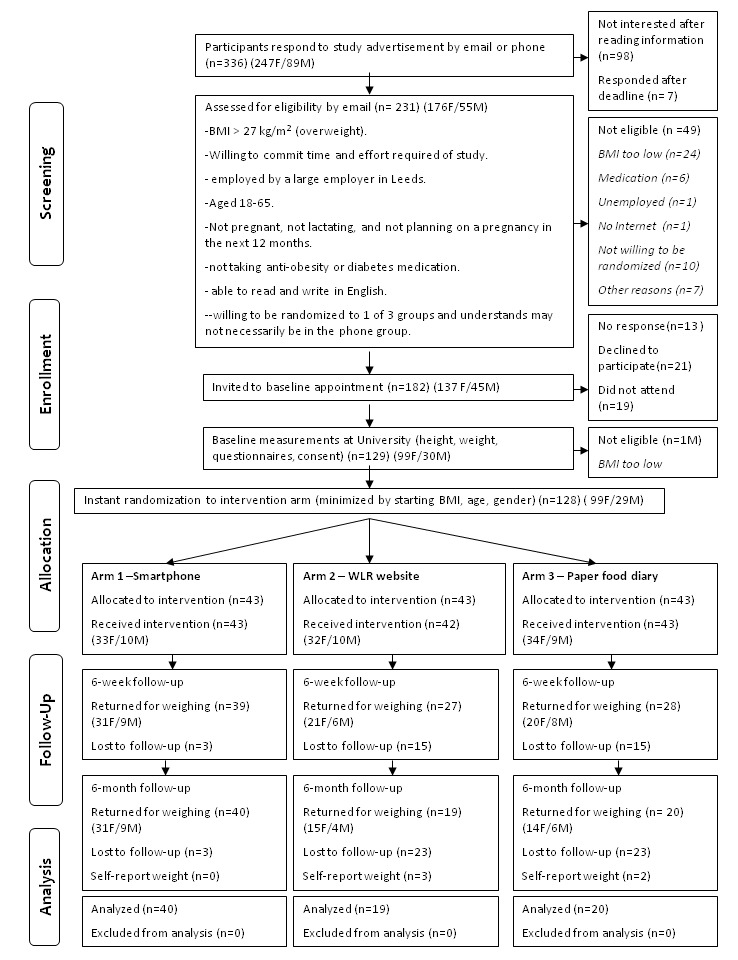
Flow of participants through a randomized, 3-armed, 6-month pilot trial of the My Meal Mate (MMM) smartphone application for weight loss (N=128).

Recruitment to the trial took 3 months between March and May 2011. Figure 3 is a CONSORT diagram [34] showing the participant flow through the trial. A total of 336 (73.5% female) people initially expressed an interest in taking part in the trial and 231 (68.8%) of these were assessed for eligibility to take part. The largest proportion (43.2%) of people who responded were from Leeds City Council, followed by the University of Leeds (27.4%). Of those screened, 49 (21.2%) were excluded for not meeting the inclusion criteria, and almost half (49.0%) were excluded because their self-reported BMI was less than 27 kg/m^2^.

In total, 182 people met the eligibility criteria and were invited to a baseline appointment. Of those invited, 21 (11.5%) declined to participate, 13 (7.1%) did not respond, and 19 (10.4%) agreed to attend but did not show up to appointments. This left 129 people who attended baseline appointments. One person was excluded at baseline because their BMI was found to be below 27 kg/m^2^. In total, 128 people were randomly allocated to 1 of the 3 groups. These 128 represented 38.1% of those who had originally expressed an interest in taking part, and 70.3% of those who had been invited to take part who met the eligibility criteria. With regard to sources of recruitment, the University of Leeds provided the most study participants (42.2%) and Leeds City Council provided the second highest proportion (39.0%). Most participants (83.6%) heard about the study from an electronic source, either by email (61.7%) or intranet (21.9%).

### Adherence to the Trial

In terms of trial retention, 94 (73.4%) people returned for 6-week follow-up measurements and 79 (61.7%) returned at 6 months. Table 2 shows the differences between those who completed 6-month follow-up compared to noncompleters. Compared to trial completers, noncompleters had a statistically significantly greater baseline BMI and BF. There was a statistically significant difference in self-reported health status at baseline between completers and noncompleters, with more completers reporting their health status as good or excellent (*P*=.001). Trial dropout was statistically significantly different among the groups (*P*=.001) with 3 people not attending 6-month follow-up in the smartphone group compared with 23 people not attending 6-month follow-up in the diary group and 23 people not attending 6-month follow-up in the website group. The reasons given for nonattendance are shown in Table 3. The most popular reasons given for nonattendance were dislike of the study equipment (n=12) and personal issues (n=6).

**Table 1:** Baseline characteristics of the participants enrolled in the 3 arms (smartphone application, website, or diary) of the 6-month pilot randomized controlled trial (N=128).

	Smartphone (n=43)	Diary (n=43)	Website (n=42)
Age (years), mean (SD)	41.2 (8.5)	42.5 (8.3)	41.9 (10.6)
Weight (kg), mean (SD)	96.4 (16.0)	97.9 (18.7)	96.4 (19.9)
Body mass index (kg/m^2^), mean (SD)	33.7 (4.2)	34.5 (5.7)	34.5 (5.6)
Body fat (%), mean (SD)	35.9 (3.8)	35.9 (4.8)	36.2 (3.9)
Gender (female), n (%)	33 (76.7)	33 (76.7)	33 (78.6)
Race/ethnicity (white), n (%)	43 (100.0)	35 (83.3)	39 (92.9)
Smoking status (current smokers), n (%)	2 (4.8)	8 (19.1)	2 (4.8)
Occupation (managerial professions), n (%)^a^	32 (74.4)	22 (51.2)	20 (48.8)
Has a university degree, n (%)	31 (72.1)	24 (55.8)	22 (52.4)
Owns a smartphone, n (%)	18 (41.9)	19 (44.2)	14 (34.2)

^a^ The occupation variable was dichotomized into managerial professions or not; it was originally measured as managerial and professional occupations, intermediate occupations, small employers and own account workers, lower supervisory and technical occupation, and semiroutine and routine occupations.

**Table 2:** Differences in baseline characteristics between trial completers and noncompleters at 6-month follow-up.

Participant characteristics	Noncompleters (n=49)	Completers (n=79)	*P* ^a^
Age (years), mean (SD)	43.2 (9.0)	41.2 (9.3)	.23
Weight (kg), mean (SD)	101.5 (18.9)	94.1 (17.1)	.03
BMI (kg/m^2^), mean (SD)	36.1 (5.8)	33.1 (4.5)	.001
Body fat (%), mean (SD)	37.4 (4.2)	35.3 (4.0)	.01
Baseline physical activity (MET-min/week), mean (SD)^b^	1468.8 (1207.9)	1638.5 (1412.3)	.67
Motivation to lose weight, mean (SD)^c^	8 (2)	8 (1)	.40
Confidence in ability to lose weight, mean (SD)^d^	7 (2)	7 (2)	.74
Number of previous weight loss attempts, mean (SD)	11.9 (16.1)	6.9 (7.9)	.11
Consideration of future consequence score, mean (SD)^e^	32.5 (8.5)	30.7 (7.2)	.22
Conscientiousness score, mean (SD)^f^	79.6 (11.8)	76.3 (11.3)	.15
Female, n (%)	38 (78)	61 (77)	.96
Obese (BMI≥30) (yes), n (%)	39 (86.7)	55 (69.6)	.05
Race/ethnicity (white), n (%)	39 (88.6)	74 (93.7)	.44
Smoking status (current smokers), n (%)	7 (15.9)	5 (6.4)	.09
Occupation (managerial professions), n (%)	21 (46.7)	50 (64.1)	.06
Reported health status as excellent or good, n (%)	26 (59.1)	68 (86.1)	.001
Main shopper (yes), n (%)	34 (75.6)	66 (83.5)	.27
Main preparer (yes), n (%)	33 (73.3)	58 (73.4)	.99
Currently dieting (yes), n (%)	31 (68.9)	59 (74.7)	.69
Constant dieter (yes), n (%)	25 (56.8)	36 (46.2)	.38
Ever kept a food diary (yes), n (%)	26 (57.8)	47 (59.5)	.85

^a^ Significant difference between completers and noncompleters assessed by 1-way *t* test.

^b^ Measured by International Physical Activity Questionnaire (IPAQ); MET-min/week=metabolic equivalent of task (MET) level × minutes of activity × events per week.

^c^ Based on a 10-point scale (1=not motivated at all; 10=extremely motivated).

^d^ Based on a 10-point scale (1=not confident at all; 10=extremely confident).

^e^ Measured by consideration of future consequences scale.

^f^ Measured by International Personality Item Pool (IPIP) conscientiousness scale.

**Table 3:** Reasons for nonattendance at 6-month follow-up for trial noncompleters.

Reason for nonattendance	Smartphone (n=43)	Diary (n=43)	Website (n=42)	Total (n=128)
Unable to contact to determine reason, n (%)	0 (0)	9 (20.9)	9 (21.4)	18 (14.1)
Did not like study equipment, n (%)	3 (6.9)	5 (11.6)	4 (9.5)	12 (9.4)
Holiday during follow-up, n (%)	0 (0)	2 (4.6)	0 (0)	2 (1.6)
Illness, n (%)	0 (0)	2 (4.6)	2 (4.7)	4 (3.1)
Personal issues, n (%)	0 (0)	3 (6.9)	3 (7.1)	6 (4.7)
Study too time-consuming, n (%)	0 (0)	1 (2.3)	1 (2.4)	2 (1.6)
Pregnancy, n (%)	0 (0)	0 (0)	1 (2.4)	1 (0.8)
Willing to self-report weight only, n (%)	0 (0)	1 (2.3)	3 (7.1)	4 (3.1)
Total	3	23	23	49

### Frequency of Use of the Interventions

**Figure 4:**
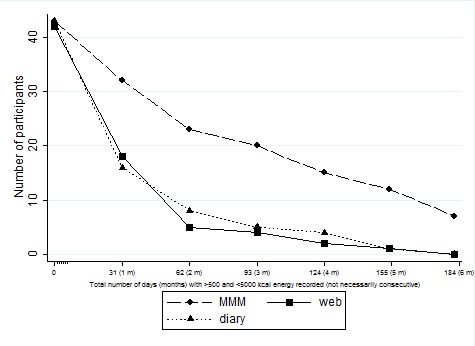
Intervention use in a randomized 3-arm pilot trial (N=128) of My Meal Mate (MMM). Adherence to the intervention arms (smartphone application, website, and diary) over the trial duration (6 months) is shown by total number of days completed in each intervention group. Data collection was conducted between May to December 2011 (total 184 days for each participant in the trial). A complete day is considered to be one with ≥500 kcal and ≤5000 kcal energy recorded. Intervention use is for overall total days completed and not necessarily consecutive days.

Table 4 shows the total number of days the interventions were used for each group at 6 weeks and 6 months (a complete day is considered as a day with ≥500 and ≤5000 kcal energy recorded). Intervention usage was highest in the smartphone group at 6 months with a mean of 92 days (SD 67) completed compared with 29 days (SD 39) in the diary group and 35 (SD 44) in the website group. There was found to be a statistically significant difference in the number of days usage among the groups at 6 weeks (*P*<.001) and 6 months (*P*=.001). Pairwise comparison showed that this difference lies between the smartphone group and the diary group (*P*<.001), between the smartphone group and the website group (*P*<.001), but not between the website group and the diary group (*P*=.14). At 6 months, 7 people had completed the smartphone electronic diary every day, no participants were found to have complete daily usage in the website and diary groups. Usage within each intervention arm declined over time as shown in Figure 4. In the smartphone group, 2 people recorded ≤7 days of food entry compared with 19 in the diary group (assuming 0 entries for 16 nonreturned diaries at 6 weeks) and 10 people in the website group. The median number of log-ins to the website over the 6-month period was 33 (interquartile range [IQR] 11-75). The frequency of website log-ins ranged from 2 to 375.

### Acceptability of Randomization and Satisfaction With Equipment

Of those who completed the 6-week questionnaires (n=93), 91.2% of smartphone participants reported that they were initially satisfied or very satisfied with their group allocation at baseline compared with 23.1% in the diary group and 71.4% in the website group (*P*=.01). When asked about how satisfied they were with the study equipment at 6 weeks, 86.8% reported that they were satisfied or very satisfied with the smartphone, compared with 57.7% in the diary group and 50.0% in the website group (*P*=.02). At 6 months, of those who completed questionnaires (n=77), 63.2% of smartphone participants were satisfied or very satisfied with the study equipment compared with 50.0% in the diary group and 42.1% in the website group (*P*=.05). At 6 months, 23 (30.0%) completers reported that they would not have volunteered for the trial if there had been no offer of using a smartphone.

No statistically significant difference was seen between the groups for self-reported ease of use of study equipment. In the smartphone group, 86.8% reported that they found their study equipment easy to use, compared with 65.0% in the diary group and 83.3% in the website group (*P*=.63). However, a statistically significant difference between the groups was found for self-reported convenience of use with 64.9% reporting that they found the smartphone convenient, (compared with 35.0% in the diary group and 52.6% in the website group, *P*<.001). In the smartphone group, 76.3% of participants agreed or strongly agreed that they felt comfortable using the study equipment to record their diet in social settings compared with 40.0% in the diary group and 21.1% in the website group (*P*<.001).

### Change in Anthropometric Measures

The pilot trial is not statistically powered to detect change in anthropometric measures; however, results are displayed to give an idea of effect size. As there is a proportion of missing data and unequal dropout, an intention-to-treat analysis was completed with baseline observations carried forward for missing data (Table 5). In the intention-to-treat analysis using all of the participants assigned to their original group, the mean weight change was –4.6 kg (95% CI –6.2 to –3.0) in the smartphone group, –2.9 kg (95% CI –4.7 to –1.1) in the diary group, and –1.3 kg (95% CI –2.7 to 0.1) in the website group. There was found to be a statistically significant difference in follow-up weight between the groups at 6 months (*P*=.004). At 6 months, weight change over time was statistically significantly greater in the smartphone group compared to the website group (–3.3 kg, 95% CI –5.4 to 1.2), but not when the smartphone group was compared to the diary group (*P*=.12).

**Table 4:** Total number of days that the interventions were used (N=128).

Intervention use	Smartphone (n=43)	Diary (n=43)	Website (n=42)	*P* ^a^
Total number of days intervention used^b^				
6 weeks (42 days), median (IQR)	36 (21-42)	29 (0-38)	15 (6-33)	.004
Completing every day, n (%)	14 (33)	8 (19)	3 (7)	
6 months (184 days), median (IQR)	82 (28-172)	18 (0-37)	15 (7-45)	<.001
Completing every day, n (%)	7 (16 )	0 (0)	0 (0)	
Completing 0 days/not returning paper diary, n (%)	1 (2)	31 (78)	3(7)	

^a^ Significant difference between groups assessed by Kruskal-Wallis equality-of-populations rank test because adherence variable not normally distributed and not improved after log transformation; significant difference at *P*<.05.

^b^ A usage day is considered to be a day with ≥500 and ≤5000 kcal energy recorded.

In the intention-to-treat analysis with baseline observation carried forward, change in BMI at 6 months was –1.6 kg/m^2^ (95% CI –2.2 to –1.1) in the smartphone group, –1.0 kg/m^2^ (95% CI –1.6 to –0.4) in the diary group, and –0.5 kg/m^2^ (95% CI –0.9 to 0.0). Change in BF was –1.3% (95% CI –1.7 to –0.8) in the smartphone group, –0.9% (95% CI –1.5 to –0.4) in the diary group, and –0.5% (95% CI –0.9 to 0.0) in the website group.

Table 6 is a subanalysis that shows the anthropometric measures for study completers only (participants who attended follow-up at 6 months). In those who completed the trial, the mean weight change was –5.0 kg (95% CI –6.7 to –3.3) in the smartphone group, –6.2 kg (95% CI –9.8 to –2.7) in the diary group, and –2.8 kg (95% CI –5.9 to 0.2) in the website group. One person allocated to the diary group reported that they had actually used a commercially available weight loss smartphone app during the trial rather than the paper diary. This person lost 32 kg overall; if they are excluded from the diary group analysis, the mean weight change in completers is –4.8 kg (95% CI –7.1 to –2.7). There were not found to be statistically significant differences in follow-up weight between the groups at 6 months (*P*=.63) or in difference in change over time (smartphone-diary, *P*=.99; smartphone-website, *P*=.40; diary-website, *P*=.47). A similar trend in results was seen for BMI and BF.

Assuming baseline observation carried forward for those who did not return for follow-up at 6 months, 35/128 (27.3% of all participants randomized) achieved a clinically significant weight loss (≥5% of initial weight). This included 16/43 participants (37.2%) in the smartphone group, 12/43 (27.9%) participants in the diary group, and 7/42 (16.7%) participants in the website group.

**Table 5:** Change in anthropometric measures using an intention-to-treat^a^ analysis.

Anthropometric measurements		Smartphone		Diary		Website		*P* ^b^
		n	Mean (95% CI)	n	Mean (95% CI)	n	Mean (95% CI)	
**Weight (kg)**								
	Baseline	43	96.8 (91.9-101.8)	43	97.9 (92.2-103.6)	42	96.4 (90.2-102.6)	
	6 weeks	43	93.9^c^ (89.0-99.0)	43	95.9^c^ (89.8-101.7)	42	95.1^c^ (89.0-101.2)	.001
	6 months	43	92.2^c^ (87.0-97.4)	43	95.0^c^ (89.0-101.0)	42	95.1 (89.0-101.3)	<.001
**BMI (kg/m^2^)**								
	Baseline	43	33.7 (32.4-35.0)	43	34.5 (32.7-36.2)	42	34.5 (32.7-36.2)	
	6 weeks	43	32.6^c^ (31.3-33.9)	43	33.7^c^ (31.9-35.5)	42	34.0 (32.3-35.7)	<.001
	6 months	43	32.1^c^ (30.7-33.5)	43	33.4 (31.5-35.4)	42	34.0 (32.3-35.8)	<.001
**Body fat (%)**								
	Baseline	42	35.9 (34.7-37.1)	42	36.0 (34.5-37.5)	42	36.3 (35.1-37.5)	
	6 weeks	42	35.0^c^ (33.7-36.2)	42	35.3^c^ (33.8-36.9)	42	36.0 (34.7-37.2)	.01
	6 months	42	34.7^c^ (33.5-35.9)	42	35.1 (33.4-36.7)	42	35.9 (34.5-37.2)	.02

^a^ The baseline measures recorded have been carried forward for missing data.

^b^ Significant difference between baseline and 6-week and 6-month follow-up assessed by paired *t* test. The regression analyses for difference in endpoints between the groups adjusts for starting weight and the 3 covariates randomized on at baseline (age, baseline BMI, and gender).

^c^ Statistically significant *P* value of <.01.

**Table 6:** Change in recorded anthropometric measures at 6 weeks (n=95) and 6 months (n=79) for trial completers.

Anthropometric measurements		Smartphone		Diary		Website		*P* ^a^
		n	Mean (95% CI)	n	Mean (95% CI)	n	Mean (95% CI)	
**Weight (kg)**								
	Baseline	40	96.8 (91.9-101.8)	20	97.9 (92.2-103.6)	19	96.4 (90.2-102.6)	
	6 months	40	92.1^b^ (86.6-97.6)	20	86.1^b^ (78.1-94.2)	19	87.0 (79.5-94.6)	.62
**BMI (kg/m^2^)**								
	Baseline	40	33.7 (32.4-35.0)	20	34.5 (32.7-36.2)	19	34.5 (32.7-36.2)	
	6 months	40	32.0^b^ (30.5-33.5)	20	30.4 (28.2-32.6)	19	31.0 (28.9-33.2)	.58
**Body fat (%)**								
	Baseline	39	35.9 (34.7-37.1)	20	36.0 (34.5-37.5)	19	36.3 (35.1-37.5)	
	6 months	39	34.6^b^ (33.4-35.9)	20	32.5 (30.1-34.8)	19	33.7 (31.7-35.8)	.89

^a^ Significant difference between baseline and 6-week and 6-month follow-up assessed by paired *t* test. The regression analysis for difference in endpoints between the groups adjusts for starting weight and the 3 covariates randomized on at baseline (age, baseline BMI, and gender).

^b^ Statistically significant *P* value of <.01.

## Discussion

This pilot trial has shown the MMM app to be a feasible and acceptable weight loss intervention.

### Recruitment and Response

In terms of recruitment and response, we were able to recruit 128 participants to the pilot, which was 95% of the original recruitment target. As is common to many weight loss trials, a large proportion of the sample (77%) were women and white (91%). The initial response rate was lower than expected and the recruitment period was extended to 3 months. Electronic media was the most successful recruitment strategy.

### Trial Retention

The pilot trial suffered from 38% attrition overall. Attrition is a serious difficulty in weight loss trials because of its potential to bias results [35]. Missing data may reflect a person’s dissatisfaction with the dietary intervention and a rebound in weight gain. To put this attrition figure into context, a systematic review of long-term weight loss trials in obese adults reported losses to follow-up in the range of 30% to 60% [36]. A review focusing specifically on Web-based interventions for weight loss found most had attrition rates greater than 20% [37]. In this trial, attrition was not equal among the groups, with more noncompleters at follow-up in the diary and Web groups compared to the smartphone group (*P*<.001). In fact, the smartphone group had extremely high retention with 93% returning for follow-up at 6 months (compared with 53% in the diary group and 55% in the website group).

Unequal dropout among groups is likely to be intervention-related [32], and a dislike of the study equipment was the most popular reason given for nonattendance at follow-up. Questionnaire data collected at follow-up also supports dissatisfaction with treatment group because at 6 weeks and 6 months satisfaction with group allocation was statistically significantly lower in the diary and Web groups. Unequal dropout is a potential source of bias in a large RCT so this will need to be considered for the full trial. Another explanation for differences in group retention may be that the smartphone group felt a greater sense of responsibility to the trial given that they had been provided with a costly piece of study equipment and had signed an agreement that they would return it. The diary and website group may have felt less obliged to return for follow-up because they did not need to physically return any equipment. This may be avoidable in a future study when it is likely that a large proportion of the population will own a smartphone (given the rising trend in smartphone ownership in the United Kingdom) so the app could be downloaded onto existing phones.

The noncompleters in the trial were more likely to have a higher BMI at baseline and report poorer health status. Other studies have shown mixed results with regard to attrition and initial body weight and a review of the behavioral approach to weight loss reports that both a higher and lower initial BMI have been linked to attrition in weight loss trials [38]. It may be that this minimal care approach is more acceptable to patients with a lower initial baseline BMI and a perception of good health, but interpretation should be cautious given the small sample size.

### Frequency of Usage of the Interventions

Adherence to dietary self-monitoring was found to be statistically significantly higher in the smartphone group than the website and paper diary group (*P*<.001). Participants were free to use the study equipment as often as they liked so the relatively high usage in the smartphone group is interesting. In all 3 groups, self-monitoring declined over time so that by 6 months only 7 participants (16% of the group) in the smartphone group had managed to record their dietary intake every day (no participants in the diary and Web group had done this). Adherence to self-monitoring is an important process outcome because it has been consistently linked to weight loss [39]. Researchers have taken different approaches to measuring adherence in studies investigating technology for weight loss so direct comparison of results is difficult.

A similar decline in adherence to dietary self-monitoring over time has been reported in other studies. In a recent RCT [13] comparing a PDA, a PDA with feedback, and a paper diary, 53% of the PDA group were adherent at 6 months compared with 60% of the PDA with feedback group and 31% of the paper diary group. Adherence was measured in that study as >50% of weekly calorie goal achieved so although the result is not directly comparable, the trend is similar. Also supporting the results of this pilot trial, the aforementioned study found that the PDA groups were statistically significantly more adherent to self-monitoring than the paper diary groups. However, in another study of dietary self-monitoring via PDA, no statistically significant difference in adherence was found between a PDA and a paper diary [40].

A key strength of this pilot is the use of a smartphone app for a high-end smartphone that is able to build on the research with PDAs (having similar self-monitoring functions) but is likely to be a more familiar technology to users. There has been a recent surge in smartphone ownership in the United Kingdom with 51% of the population reporting to own a smartphone [41]. It is evident that there is consumer demand for diet tracking apps due to the popularity of commercial systems such as MyFitnessPal [21] and Lose It! [42]. Investigating a researcher-developed app gives a unique opportunity to collect data on usage directly from the participants. In terms of acceptability, MMM was more highly rated in comparison to the diary and website on a range of acceptability measures, including overall satisfaction, convenience, and acceptability of use in social settings.

### Weight Loss

Although the pilot trial was not statistically powered to detect a difference in weight change among the groups, it has provided some data on effect size. Completers in the smartphone group had a mean weight loss of -5.0 kg (95% CI -6.7 to -3.3) after 6 months. This is comparable to the weight loss achieved in a large multicentered RCT of popular commercial diet programs that reported an average weight loss of -5.9 kg at 6 months across all diets [43]. The diary and website group had a comparable mean weight change at 6 months as those who returned for 6-month follow-up. When an intention-to-treat analysis is used with baseline observation carried forward for missing data, the mean weight change in the diary and Web groups is more modest.

### Strengths

This pilot trial has several strengths including its randomized design. Although researchers have investigated dietary self-monitoring as an adjunct or follow-up to a behavioral weight loss intervention [44] or used a smartphone app to enhance adherence to another intervention [45], this pilot trial has taken a minimal contact approach with no dietary advice at baseline. The weight loss seen in the smartphone arm is encouraging given that a minimal contact approach could be a cost-effective and wide-reaching strategy. This approach could also be especially beneficial to those who would prefer not to attend face-to-face meetings. Another strength of the trial is the up-to-date app for tracking diet and physical activity which is comparable in appearance and functionality to commercial diet tracking apps. Despite their apparent popularity, these commercial apps have not been comprehensively evaluated to date.

### Limitations

Generalizability of the pilot results is limited given that the sample are predominantly white, female, and employed in managerial/professional occupations. The MMM app was a prototype app and participants reported that they frequently encountered bugs that caused the app to close. This may have affected participant engagement. Twenty people in the trial also reported that they had used another intervention (either instead of or in addition to their originally allocated intervention) during the trial. Seven participants from the smartphone group reported using a weight loss website, 7 people from the diary group reporting using a website, and 4 using a smartphone app and 2 from the website group reported to have used a smartphone app. One participant originally randomized to the diary group enjoyed self-monitoring but wanted to make it more convenient, so downloaded the commercially available MyFitnessPal app and used this for the duration of the trial. This person went on to lose 32 kg and had a strong influence on the mean weight change seen in the diary group. The degree of contamination seen in the trial is a serious issue and has implications for the design of a definitive RCT. In the pilot trial, participants knew what interventions were available in the trial and although they had all agreed to sign up with the understanding that they would be randomized to a group and not necessarily receive the intervention of their choice, it is a possibility that the trial raised their awareness of newer ICT-based methods of weight loss which they may not have already been aware of. In a definitive trial, the design would need to be altered to address contamination. A delayed control may be used so that participants in the control group could be asked not to use other weight management interventions during the trial and participants would be recruited in such a way that did not reveal what other groups were receiving.

### Implications

Further analysis will be performed on the pilot data to investigate the characteristics of successful users in the trial to see if there is any scope for tailoring this approach. Given that some participants have more success in behavioral weight loss programs than others [38], knowing who will do well with this smartphone approach is key to tailoring it appropriately. This pilot trial has several implications for a future trial. Given the unequal dropout in the comparator group, a larger trial may need to consider what if any retention strategies are appropriate. Two control groups were used in the pilot, but because participants had comparable adherence and weight loss in the diary group this may be the most cost-effective for a full trial. Further research would also benefit from an economic analysis to investigate the cost of implementing a smartphone app intervention compared with other types of weight management intervention.

### Conclusion

This pilot trial of a smartphone app for weight loss has shown that it is both an acceptable and feasible intervention. Adherence to the intervention and to the trial was greater in the smartphone group than the comparator groups and the app was rated highly in satisfaction and acceptability. To our knowledge, there have been no large RCTs of smartphone apps for weight loss and this pilot trial provides valuable data that could be used to inform such a trial.

## Multimedia Appendix 1

CONSORT EHEALTH checklist V1.6.2 [33].

## Abbreviations

ANOVA: analysis of variance
BF: body fat
BMI: body mass index
ICT: information and communication technology
IPIP: International Personality Item Pool
MET: metabolic equivalent of task
MMM: My Meal Mate
NHS: National Health Service
PDA: personal digital assistants
PmEB: Patient-Centered Assessment and Counseling Mobile Energy Balance
RCT: randomized controlled trial
SMS: short message service
