# Supplementary material for: Adherence to a Smartphone Application for Weight Loss Compared to Website and Paper Diary: Pilot Randomized Controlled Trial
Source: J Med Internet Res. 2013 Apr 15;15(4):e32. doi: 10.2196/jmir.2283 (PMC3636323; doi:10.2196/jmir.2283)
Supplement: Supplementary file 1 [file jmir_v15i4e32_app1.pdf]

CONSORT-EHEALTH Checklist V1.6.2 Report

(based on CONSORT-EHEALTH V1.6), available at [http://tinyurl.com/consort-ehealth-v1-6].

2283

Date completed

12/6/2012 10:40:45

by

Michelle

Results of a pilot randomised controlled trial of “My Meal Mate”
(MMM): a smartphone application for weight loss

TITLE

1a-i) Identify the mode of delivery in the title

Results of a pilot "randomised controlled trial" of “My Meal Mate”
(MMM): a "smartphone application" for weight loss

1a-ii) Non-web-based components or important co-interventions in title

1a-iii) Primary condition or target group in the title

a smartphone application "for weight loss"

ABSTRACT

1b-i) Key features/functionalities/components of the intervention and comparator in the METHODS section of the ABSTRACT

Methods
A sample of 128 overweight volunteers were randomised to receive a "weight management intervention either delivered by smartphone app (MMM), website or paper diary". Participants tested the intervention for 6 months and anthropometric and questionnaire data was collected at baseline, 6 weeks and 6 months.

1b-ii) Level of human involvement in the METHODS section of the ABSTRACT

1b-iii) Open vs. closed, web-based (self-assessment) vs. face-to-face assessments in the METHODS section of the ABSTRACT

1b-iv) RESULTS section in abstract must contain use data

1b-v) CONCLUSIONS/DISCUSSION in abstract for negative trials

INTRODUCTION

2a-i) Problem and the type of system/solution

"Associated with a range of serious and difficult to treat conditions such as diabetes, some cancers and heart disease, obesity is estimated by the World Health Organisation to be the fifth leading risk for global deaths[1]. In the UK, obesity is a major public health concern reported to affect a quarter of the adult population[2]. The economic burden to the NHS is significant, with the direct cost of spending on overweight and obesity estimated at $4.2 billion in 2007[3]. The effective treatment of obesity and overweight is challenging and NHS primary care struggles to provide effective support to meet demand[4]. A large community based survey showed that individuals desire alternatives to face to face weight loss treatments and if given the choice some would be interested in engaging with minimal contact weight management programmes[5].

Information communication technology (ICT) based weight management interventions provide an opportunity to engage a wide audience in a potentially flexible and cost effective way. In recent years, research into mobile devices to facilitate dietary and physical activity self monitoring and weight related behavior change has grown."

2a-ii) Scientific background, rationale: What is known about the (type of) system

The comparator group was chosen as a "standard care" no intervention control is very difficult within the UK as NHS primary care approach to obesity varies by GP practices. As a pilot trial, we decided to have two comparison arms involving self monitoring of diet and activity in order to inform on the most appropriate comparison group for the definitive trial.

"Although there have been RCTs using text messaging interventions for weight management, personal digital assistants for self monitoring and smartphone apps as adjuncts to other weight management interventions, to our knowledge. There have been no RCTs of a smartphone app as a weight loss intervention in itself using both self monitoring and text messaging functions. A trial of this type is necessary as smartphone apps are readily available to the public to download and likely to be used as a “stand alone” intervention rather than as an adjunct to another intervention (such as podcasts or face to face advice). "

METHODS

3a) CONSORT: Description of trial design (such as parallel, factorial) including allocation ratio

"The aim of this pilot is to test the acceptability and feasibility (including; recruitment, drop-out and adherence) of MMM with a view to informing a larger trial."

3b) CONSORT: Important changes to methods after trial commencement (such as eligibility criteria), with reasons

No changes to methods after trial commencement.

3b-i) Bug fixes, Downtimes, Content Changes

4a) CONSORT: Eligibility criteria for participants

"The eligibility criteria was a body mass index (BMI)≥27kg/m2 ; aged 18-65; willing to commit the necessary time and effort to the study; employed by a large employer in Leeds; not pregnant, breast-feeding or planning a pregnancy; not taking anti-obesity medication or medication/insulin for diabetes; not had surgery for weight loss; not taking the antidepressant sertraline (due to associations with weight gain); able to read and write in English; able to access the internet; willing to be randomised to one of three groups. An inclusion cut-off BMI of ≥ 27kg/m2 as opposed to the more familiar cut-off point of 25kg/m2 was chosen in order to ensure that participants had a reasonable amount of weight to lose in 6 months before maintenance of weight loss and also as a safety measure so that they would be unlikely to lose so much weight that they fell below the defined “healthy” BMI range given that the app would be used for 6 months without any clinical supervision."

4a-i) Computer / Internet literacy

4a-ii) Open vs. closed, web-based vs. face-to-face assessments:

"Participants were recruited from large employers within Leeds, UK, by advertising via email; intranet; posters and newsletters. Advertising material encouraged participants to contact the research team, following which they were emailed information sheets and an eligibility questionnaire."

"After randomisation, groups of participants were taken to separate rooms to receive standardised training in their allocated study equipment. Participants were instructed to use the study equipment every day for a week and then to use it as much as they desired over the trial period. The smartphone group were given a "HTC Desire" smartphone with the MMM pre-downloaded, the website group were given a voucher providing 6 months access to the WLR website and the food diary group were given a paper food diary, calorie counting book and calculator. All participants were given access to an internet forum for social support."

4a-iii) Information giving during recruitment

4b) CONSORT: Settings and locations where the data were collected

"Eligible participants were invited to attend a baseline enrolment session at the University of Leeds, where height, weight and percentage body fat were measured by research assistants, and baseline questionnaires completed."

4b-i) Report if outcomes were (self-)assessed through online questionnaires

There were no online questionnaires, all of the questionnaires were paper based.

4b-ii) Report how institutional affiliations are displayed

5) CONSORT: Describe the interventions for each group with sufficient details to allow replication, including how and when they were actually administered

5-i) Mention names, credential, affiliations of the developers, sponsors, and owners

5-ii) Describe the history/development process

5-iii) Revisions and updating

5-iv) Quality assurance methods

5-v) Ensure replicability by publishing the source code, and/or providing screenshots/screen-capture video, and/or providing flowcharts of the algorithms used

5-vi) Digital preservation

5-vii) Access

The smartphone application was pre-downloaded onto the smartphone for participants by the research team and the participants were given a smartphone to use in the trial. For the slimming website, participants were provided with a log-in to use the website during the trial. The participants were not given any money to take part.

"The smartphone group were given a "HTC Desire" smartphone with the MMM pre-downloaded, the website group were given a voucher providing 6 months access to the WLR website and the food diary group were given a paper food diary, calorie counting book and calculator. All participants were given access to an internet forum for social support."

5-viii) Mode of delivery, features/functionalities/components of the intervention and comparator, and the theoretical framework

The following behaviour change techniques were used in the interventions; goal setting, self monitoring (of diet, physical activity and weight), feedback (instant nutritional feedback and by text message). The feedback was designed to enhance self-efficacy and encourage participants to reherse their goal.

"Current UK evidence based obesity guidelines advocate a "lifestyle change" approach to treatment[20] so in line with this the key behavioural strategies of goal setting, self monitoring and feedback underpin the MMM app. MMM allows system users to set a weight loss goal and self monitor daily calorie intake towards achieving the goal. Users select food and drink consumed from a database and log items in an electronic food diary. Physical activity can also be recorded in the diary enabling the user to receive instant feedback on their energy expenditure. Progress is tracked graphically and further support is provided via tailored weekly text messages. A library of text messages was created and each message was triggered according to progress towards the users' calorie targets. The messages aimed to enhance the users self efficacy by encouraging the user to rehearse their weight loss goal and reinforce positive behavioural beliefs (about competence, confidence and mastery)."

5-ix) Describe use parameters

5-x) Clarify the level of human involvement

5-xi) Report any prompts/reminders used

Participants received feedback by weekly text message.

"A library of text messages was created and each message was triggered according to progress towards the users' calorie targets. The messages aimed to enhance the users self efficacy by encouraging the user to rehearse their weight loss goal and reinforce positive behavioural beliefs (about competence, confidence and mastery)."

5-xii) Describe any co-interventions (incl. training/support)

Participants in all three arms received a 30 minute training session at the beginning of the trial. Ths involved a demonstration of how to use the study equipment to set a weight loss goal and self monitor their diet.

"After randomisation, groups of participants were taken to separate rooms to receive standardised training in their allocated study equipment. Participants were instructed to use the study equipment every day for a week and then to use it as much as they desired over the trial period."

6a) CONSORT: Completely defined pre-specified primary and secondary outcome measures, including how and when they were assessed

As a pilot trial, the primary outcomes were feasibility and acceptability. Feasibility was measured by numbers completing the trial and number of days participants self monitored diet. Acceptability was assessed with Likert scales administered at the end of the trial. Secondary outcomes of weight, height and % fat mass were objectively measured by researchers at baseline, 6 weeks and 6 months.

"This trial is a pilot aiming to collect acceptability and feasibility outcomes of a minimal contact, self-monitoring weight management intervention delivered by a smartphone app."

"The questionnaires were designed to collect information on; demographics, technology usage, attitudes towards weight loss, physical activity[23], eating behaviour[24] and a variety of psychosocial variables[25, 26]. Weight (without shoes) and body fat (%) were measured using "Weight Watchers 8958U: Body Analyser scale" portable weighing scales. Height (without shoes) was measured using a portable stadiometer to the nearest 0.1cm."

6a-i) Online questionnaires: describe if they were validated for online use and apply CHERRIES items to describe how the questionnaires were designed/deployed

6a-ii) Describe whether and how "use" (including intensity of use/dosage) was defined/measured/monitored

6a-iii) Describe whether, how, and when qualitative feedback from participants was obtained

6b) CONSORT: Any changes to trial outcomes after the trial commenced, with reasons

None

7a) CONSORT: How sample size was determined

7a-i) Describe whether and how expected attrition was taken into account when calculating the sample size

7b) CONSORT: When applicable, explanation of any interim analyses and stopping guidelines

None

8a) CONSORT: Method used to generate the random allocation sequence

"After measurements had been taken participants were randomised by a process of minimisation using the software package "minim"[27] to one of three groups. The minimisation balanced equally at the medians on three factors; starting BMI, age and gender. Minimisation was used as this method has the advantage over simple or stratified randomisation of providing very similar balanced groups in small samples[28]."

8b) CONSORT: Type of randomisation; details of any restriction (such as blocking and block size)

"After measurements had been taken participants were randomised by a process of minimisation using the software package "minim"[27] to one of three groups. The minimisation balanced equally at the medians on three factors; starting BMI, age and gender. Minimisation was used as this method has the advantage over simple or stratified randomisation of providing very similar balanced groups in small samples[28]."

9) CONSORT: Mechanism used to implement the random allocation sequence (such as sequentially numbered containers), describing any steps taken to conceal the sequence until interventions were assigned

"After measurements had been taken participants were randomised by a process of minimisation using the software package "minim"[27] to one of three groups. The minimisation balanced equally at the medians on three factors; starting BMI, age and gender. Minimisation was used as this method has the advantage over simple or stratified randomisation of providing very similar balanced groups in small samples[28]."

10) CONSORT: Who generated the random allocation sequence, who enrolled participants, and who assigned participants to interventions

An independent researcher sat in a different room to allocate the participants using a computer program called "minim". The researcher responsible for randomisation did not have any contact with the participants and assigned participants to a group A, B or C based on ID number (and age, gender and weight) but did not know which groups the letters corresponded to.

11a) CONSORT: Blinding - If done, who was blinded after assignment to interventions (for example, participants, care providers, those assessing outcomes) and how

11a-i) Specify who was blinded, and who wasn't

The participants were not blinded however the researchers who weighed the participants and adminstered questionnaires were blinded at baseline and at follow up.

11a-ii) Discuss e.g., whether participants knew which intervention was the "intervention of interest" and which one was the "comparator"

11b) CONSORT: If relevant, description of the similarity of interventions

The same behaviour change techniques are used in the comparison interventions.

"The comparison interventions provided an opportunity to deliver a similar self monitoring intervention by different mediums as each provides goal setting, and self monitoring using the same "Weight Loss Resources" food database."

12a) CONSORT: Statistical methods used to compare groups for primary and secondary outcomes

"Statistical analysis was carried out using Stata 11 (Stata corp). As this is a pilot trial and not powered to detect weight change, most analysis is descriptive. The effectiveness of the minimisation procedure was assessed by determining baseline balance between the groups. Where analysing differences between the three intervention groups, oneway anova was used for continuous outcomes found to be normally distributed or kruskal wallis where not normally distributed. For the analysis of completers vs. non completers, t-tests were used for continuous outcomes which were normally distributed and the Wilcoxon rank sum test for non normally distributed outcomes. Differences between groups for categorical data were analysed using chi squared tests."

12a-i) Imputation techniques to deal with attrition / missing values

"As there is a proportion of missing data and unequal drop-out between groups, two analyses have been conducted; an intention to treat analysis in which all are included but using baseline weight carried forward for missing data and an analysis in just those who completed 6 month follow up."

12b) CONSORT: Methods for additional analyses, such as subgroup analyses and adjusted analyses

"The pilot trial is not powered to detect change in anthropometric measures however results are displayed for interest and to provide information on effect size. A regression analysis was used to test between group difference in change in anthropometric measures adjusting for the three factors used in randomisation at baseline (age, gender and starting BMI). "

RESULTS

13a) CONSORT: For each group, the numbers of participants who were randomly assigned, received intended treatment, and were analysed for the primary outcome

A consort diagram is included in the paper with this data.

Number randomly assigned - 128
Number received intended treatment - 128
Number analysed - 79

13b) CONSORT: For each group, losses and exclusions after randomisation, together with reasons

smartphone group loss to follow up - 3/43
website group loss to follow up - 23/42
paper diary group loss to follow up - 23/43

13b-i) Attrition diagram

14a) CONSORT: Dates defining the periods of recruitment and follow-up

Recruitment began April 2011
Follow up was during November-December 2011

"Recruitment to the trial took three months."

14a-i) Indicate if critical “secular events” fell into the study period

14b) CONSORT: Why the trial ended or was stopped (early)

The trial was funded for 6 months and ran for 6 months.

15) CONSORT: A table showing baseline demographic and clinical characteristics for each group

This is included in the paper.

15-i) Report demographics associated with digital divide issues

The baseline characteristic table addresses; age, gender, occupation, BMI, body fat, smartphone ownership, education, ethnicity, smoking status.

16a) CONSORT: For each group, number of participants (denominator) included in each analysis and whether the analysis was by original assigned groups

16-i) Report multiple “denominators” and provide definitions

All participants were analysed in their assigned groups. One analysis used baseline observation carried forward and one using completers only. Numbers analysed are shown in the participant flow diagram and in the results tables.

16-ii) Primary analysis should be intent-to-treat

17a) CONSORT: For each primary and secondary outcome, results for each group, and the estimated effect size and its precision (such as 95% confidence interval)

Effect size and 95% confidence intervals are presented in the results tables.

17a-i) Presentation of process outcomes such as metrics of use and intensity of use

17b) CONSORT: For binary outcomes, presentation of both absolute and relative effect sizes is recommended

not applicable.

18) CONSORT: Results of any other analyses performed, including subgroup analyses and adjusted analyses, distinguishing pre-specified from exploratory

"
The pilot trial is not statistically powered to detect change in anthropometric measures however results are displayed for interest and to provide information on effect size. A regression analysis was used to test between group difference in change in anthropometric measures adjusting for the three factors used in randomisation at baseline (age, gender and starting BMI). As there is a proportion of missing data and unequal drop-out between groups, two analyses have been conducted; an intention to treat analysis in which all are included but using baseline weight carried forward for missing data and an analysis in just those who completed 6 month follow up."

18-i) Subgroup analysis of comparing only users

19) CONSORT: All important harms or unintended effects in each group

None

19-i) Include privacy breaches, technical problems

19-ii) Include qualitative feedback from participants or observations from staff/researchers

DISCUSSION

20) CONSORT: Trial limitations, addressing sources of potential bias, imprecision, multiplicity of analyses

20-i) Typical limitations in ehealth trials

"Generalisability of the pilot results is limited given that the sample are predominantly white, female and employed in managerial/professional occupations. A limitation of this pilot trial is that that MMM was a prototype app and participants reported that they frequently encountered bugs which caused the app to close. This may have affected participant engagement. Twenty people in the trial also reported that they had used another intervention (either instead of or as well as their originally allocated intervention) during the trial. Seven participants from the smartphone group reported using a slimming website, 7 people from the diary group reporting using a website and 4 using a smartphone app and 2 from the website group reported to have used a smartphone app. One participant originally randomised to the diary group reported to enjoy self monitoring but wanted to make it more convenient so downloaded the commercially available “my fitness pal” and used this for the duration of the trial. This person went on to lose 32kg and has had a strong influence on the mean weight change seen in the diary group. The degree of contamination seen in the trial is a serious issue and has implications for the design of a definitive RCT. In the pilot trial, participants knew what interventions were available in the trial and although they had all agreed to sign up with the understanding that they would be randomised to a group and not necessarily receive the intervention of their choice it is a possibility that the trial raised their awareness of newer ICT based methods of weight loss which they may not have already been aware of. In a definitive trial, the design would need to be altered in order to address contamination. A delayed control may be used so that participants in the control group could be asked not to use other weight management interventions during the trial and participants would be recruited in such a way that did not reveal what other groups were receiving.”

21) CONSORT: Generalisability (external validity, applicability) of the trial findings

**21-i) Generalizability to other populations**
**21-ii) Discuss if there were elements in the RCT that would be different in a routine application setting**
**22) CONSORT: Interpretation consistent with results, balancing benefits and harms, and considering other relevant evidence**
**22-i) Restate study questions and summarize the answers suggested by the data, starting with primary outcomes and process outcomes (use)**
"This pilot trial has shown “My Meal Mate” (MMM) to be a feasible and acceptable weight loss intervention.<br>Recruitment and response<br>In terms of recruitment and response, we were able to recruit 128 participants to the pilot which was 95% of the original recruitment target. As is common to many weight loss trials, a large proportion of the sample (77%) were women and of white ethnic origin (91%). The initial response rate was lower than expected and the recruitment period was extended to three months. Electronic media was the most successful recruitment strategy.<br><br>Trial retention<br>The pilot trial suffered from 38% attrition overall. Attrition is a serious difficulty in weight loss trials due to its potential to bias results[30]. Missing data may reflect a person’s dissatisfaction with the dietary intervention and a rebound in weight loss. To put this attrition figure into context, a systematic review of long term weight loss trials in obese adults, reported losses to follow up in the range of 30-60%[31]. A review focussing specifically on web-based interventions for weight loss found most had attrition rates greater than 20%[32]. In this trial, attrition was not equal between the groups, with more non-completers at follow up in the diary and web group compared to the smartphone group (P = <0.0001). In fact, the smartphone group had extremely high retention with 93% returning for follow up at 6 months (compared to 53% in the diary group and 55% in the website group).<br>Unequal drop-out between groups is likely to be intervention related[28] and a dislike of the study equipment was the most popular reason given for non attendance at follow up. Questionnaire data collected at follow-up also supports dissatisfaction with treatment group as at 6 weeks and 6 months satisfaction with group allocation was statistically significantly lower in the diary and web groups. Unequal drop-out is a potential source of bias in a large RCT so this will need to be considered for the full trial. Another explanation for differences in group retention may be that the smartphone group felt a greater sense of responsibility to the trial given that they had been provided with a costly piece of study equipment and had signed an agreement that they would return it. The diary and website group may have felt less obliged to return for follow up as they did not need to physically return equipment. This may be avoidable in a future study when it is likely that a large proportion of the population will own a smartphone (given the rising trend in smartphone ownership in the UK) so the app could be downloaded onto existing phones.<br>The non-completers in the trial were more likely to have a higher BMI at baseline and report poorer health status. Other studies have shown mixed results with regard to attrition and initial body weight and a review of the behavioural approach to weight loss reports that both a higher and lower initial BMI have been linked to attrition in weight loss trials[33]. It may be that this minimal care approach is more acceptable to patients with a lower initial baseline BMI and a perception of good health but interpretation should be cautious given the small sample size.<br><br>Frequency of usage of the interventions<br>Adherence to dietary self monitoring was found to be statistically significantly higher in the smartphone group than the website and paper diary group (P =<0.001). Participants were free to use the study equipment as often as they liked so the relatively high usage in the smartphone group is interesting. In all three groups, self monitoring declined over time so that by 6 months only a small number7 participants (16% of the group) in the smartphone group had managed to record their dietary intake every day. Adherence to self monitoring is an important process outcome as it has been consistently linked to weight loss[34]. Researchers have taken different approaches to measuring adherence in studies investigating technology for weight loss so direct comparison of results is difficult.<br>A similar decline in adherence to dietary self monitoring over time has been reported in other studies. In a recent RCT[12] comparing a PDA, PDA with feedback and a paper diary, 53% of the PDA group were adherent at 6 months, 60% of the PDA with feedback group and 31% of the paper diary group. Adherence was measured in that study as >50% of weekly calorie goal achieved so although the result is not directly comparable, the trend is similar. Also supporting the results of this pilot trial, the aforementioned study found that the PDA groups were statistically significantly more adherent to self monitoring than the paper diary groups. However, in another study of dietary self monitoring via PDA, no statistically significant difference in adherence was found between a PDA and a paper diary[35].<br>A key strength of this pilot is the use of a smartphone app for a high end smartphone which is able to build on the research with PDAs (having similar self monitoring functions) but is likely to be a more familiar technology to users. There has been a recent surge in smartphone ownership in the UK with 51% of the population reporting to own a smartphone[36]. It is evident that there is consumer demand for diet tracking apps due to the popularity of commercial systems such as “my fitness pal”[18] and “lose-it”[37]. Investigating a researcher developed app gives a unique opportunity to collect data on usage directly from the participants. In terms of acceptability, MMM was more highly rated in comparison to the diary and website on a range of acceptability measures including overall satisfaction, convenience and acceptability of use in social settings.<br><br>Weight loss<br>Although the pilot trial was not statistically powered to detect a difference in weight change between the groups it has provided some data on effect size. Completers in the smartphone group had a mean weight loss of - 5.0kg (95% CI: - 6.7kg to -3.3kg) after 6 months. This is comparable to the weight loss achieved in a large multi-centred RCT of popular commercial diet programmes which reported an average weight loss of - 5.9kg at 6 months across all diets[38]. The diary and website group had a comparable mean weight change at 6 months in those that returned for 6 month follow up. When an intention to treat analysis is used with baseline observation carried forward for missing data the mean weight change in the diary and web groups is more modest."
**22-ii) Highlight unanswered new questions, suggest future research**
Other information
**23) CONSORT: Registration number and name of trial registry**
ClinicalTrials.gov.<br><br>registration number; NCT01744535
**24) CONSORT: Where the full trial protocol can be accessed, if available**
Not available online
**25) CONSORT: Sources of funding and other support (such as supply of drugs), role of funders**
Funded by National Prevention Research Initiative (G0802108).
**X26-i) Comment on ethics committee approval**
**x26-ii) Outline informed consent procedures**
**X26-iii) Safety and security procedures**

X27-i) State the relation of the study team towards the system being evaluated
